# Phone Messaging to Prompt Physical Activity and Social Support Among Low-Income Latino Patients With Type 2 Diabetes: A Randomized Pilot Study

**DOI:** 10.2196/diabetes.7063

**Published:** 2017-06-06

**Authors:** Magaly Ramirez, Shinyi Wu

**Affiliations:** 1 Fielding School of Public Health Department of Health Policy and Management University of California, Los Angeles Los Angeles, CA United States; 2 Suzanne Dworak-Peck School of Social Work University of Southern California Los Angeles, CA United States; 3 Viterbi School of Engineering Epstein Department of Industrial and Systems Engineering University of Southern California Los Angeles, CA United States; 4 Edward R. Roybal Institute on Aging University of Southern California Los Angeles, CA United States

**Keywords:** short message service, reminder system, pilot project, exercise, Hispanic Americans, type 2 diabetes mellitus, self-care, social support

## Abstract

**Background:**

Given disparities in diabetes prevalence, receipt of diabetes education, diabetes knowledge, and self-management behaviors among Latinos, there is a need to provide education and ongoing support to this population. Phone-based interventions have the potential to reach and engage both patients and their family members and friends.

**Objective:**

The aim of this study was to investigate the feasibility, perceived usefulness, and potential effectiveness of a short text or voice message (STVM) intervention to activate (1) physical activity (PA) behavior change among urban, low-income Latino adults with type 2 diabetes and (2) supportive behaviors by their family members and friends.

**Methods:**

A 12-week pilot study randomized 42 participants recruited in person from a safety-net ambulatory care clinic in Los Angeles into one of the 3 study arms: control, phone messaging (PM), and phone messaging plus social support from family members and friends (PM+FF). All participants were prompted to set PA goals and to self-monitor PA behavior using pedometers and walking logs. PM and PM+FF participants received STVMs with reminders to review goals and self-monitor, PA behavior change education, and feedback on performance. Participants in the PM+FF arm also had their family members and friends receiving STVMs with suggestions for how they could support the participant’s PA behavior change efforts. Participants completed semistructured assessments in person at baseline, 6 weeks, and 12 weeks. Outcomes were PA (steps/day) and perceived social support from family members and friends.

**Results:**

Among PM and PM+FF participants, those who opted to receive text messages (short message service, SMS) responded to 62.7% (128/204) of SMS text messages requiring a response while those who opted to receive voice messages responded 30% (12/40) of the time. Participants perceived guidance in self-regulation as useful, particularly self-monitoring, goal setting, self-instruction, feedback, and social support. All participants increased PA at 6 weeks, but only the PM and PM+FF arms increased PA at 12 weeks. All study arms experienced an increase in perceived social support from family members and friends at 6 weeks, but only those in the PM+FF arm had an increase in the perception of social support at 12 weeks.

**Conclusion:**

Designing an STVM intervention based on self-regulation techniques is feasible and perceived as useful by participants. The STVM intervention has the potential to improve PA in terms of daily steps and perceived social support from family members and friends.

**Trial Registration:**

Clinicaltrials.gov NCT02850770; https://clinicaltrials.gov/ct2/show/NCT02850770 (Archived by WebCite at http://www.webcitation.org/query?id=1495567756845570)

## Introduction

Latinos in the United States have a 66% higher risk of developing diabetes and are 1.5 times more likely to die from the disease compared with non-Latino whites [1]. Education can effectively prepare individuals with knowledge, skills, and abilities necessary to perform the diabetes self-care behaviors that will improve glycemic control and reduce the risk of complications [2-5], but Latinos are less likely to receive education compared with non-Latino whites [6]. Deficiencies in knowledge about diabetes have been observed among low-income, predominantly Latino patients in a safety-net health care setting [7]. Additionally, compared with non-Latino whites, Latinos report worse engagement in diabetes self-care behaviors [6]. For example, a significantly lower proportion of Mexican Americans than whites with type 2 diabetes (T2D) report getting the recommended levels of physical activity (PA) (28% vs 32%, respectively) [8].

Given the aforementioned disparities among Latinos with diabetes, there is a need to provide education and ongoing support to this population. Automated text and voice messaging has enormous potential to target all aspects of diabetes self-management and lifestyle behavior modification and reach many people at a relatively low cost [9]. In theory, these tools can promote effective self-care behaviors, assist with monitoring changes in health and health behaviors, and enhance communication between patients and potential supports for their diabetes self-management [10]. A systematic review of reviews found that SMS text-messaging (short message service, SMS) interventions significantly improve health outcomes and health behaviors of individuals with diabetes [9]. The interventions targeted multiple aspects of diabetes self-management at once, but tended to focus on blood glucose monitoring. Lifestyle behavior modification, such as engagement in PA, which Latinos perceive as one of the most difficult aspects of diabetes self-care [11], perhaps because of a lack of behavioral activation or knowledge about its importance [7], were generally not emphasized. Extensive evidence shows that PA plays an important role in risk reduction and control of T2D [12-18]. In addition, only 1 of 16 reviewed interventions for individuals with diabetes included Latinos. The only study, led by Arora et al, found that a unidirectional text-messaging intervention targeting Latinos with poorly controlled diabetes in an emergency department did not produce significant improvements in blood glucose [19,20]. Perhaps these findings were observed because of the study population (emergency department patients with poorly controlled diabetes represent a high-risk group [21]) or because of the intervention design (it did not incorporate interactive elements such as assessments and feedback [22]). Overall, there is limited evidence for the feasibility, acceptance, and effectiveness of interactive text and voice messaging interventions to promote PA among Latino adults with T2D in order to improve their diabetes outcomes.

In addition to text and voice messaging, another potential source of education and ongoing support are family members and friends. A qualitative study found that Latino adults with diabetes desired an increase in support from family members, and that family members were eager to provide support, but did not know how [11]. Family members of low-income, predominantly Latino patients with diabetes in a safety-net health care setting have demonstrated poor knowledge about diabetes [7]. Text and voice messaging interventions may leverage on the willingness of family members and friends to learn about the disease and how to provide support. In an intervention to support diabetes self-management among Spanish-speaking patients, family members and friends were called and emailed to get notified of patients’ health status and receive instructions for how to provide support [23,24], but the impact of their support on health outcomes or health behaviors was not reported. In general, it is unknown whether it is feasible to reach and engage family members and friends through technology-based interventions and what is the impact on patient outcomes.

Furthermore, few text-messaging interventions to promote healthy behaviors are designed with reference to behavior change theory [9]. Theoretically informed interventions are expected to be more effective than those that are not as they target causal determinants of behavior and behavior change [25]. In this study, we designed a short text or voice message (STVM) intervention targeting both Latino adults with T2D and their family members and friends. The intervention was based self-regulation techniques derived from the Social Cognitive Theory (SCT) [26] and patient preferences for how to operationalize these techniques [27]. The techniques were as follows: prompting goal setting, prompting self-monitoring, providing feedback on performance, and prompting social support. SCT posits that a person can achieve self-regulation by observing the behavior, identifying attainable short- and long-term behavior change goals, receiving information about the recorded behavior, and receiving encouraging support from another person. Meta-regression analyses of interventions to promote PA and healthy eating indicate these self-regulation techniques are associated with positive outcomes [28,29].

Given the potential of phone-based interventions to reach and engage both patients and family members and friends [9,10,23,24], the objective of this study was to investigate the feasibility, perceived usefulness, and potential effectiveness of the STVM intervention that was designed based on self-regulation techniques and patient preferences to activate (1) PA behavior change (steps/day) among urban, low-income Latino adults with T2D and (2) supportive behaviors by family members and friends.

## Methods

### Ethical Considerations

The Health Sciences Institutional Review Board at the University of Southern California approved all study procedures. Eligible participants provided written informed consent.

### Study Design

We conducted a pilot study (ClinicalTrials.gov NCT02850770) that randomized participants into one of three study arms: control, phone messaging (PM), and phone messaging plus social support from family members and friends (PM+FF). The PM versus control comparison was intended to provide insight into the potential effectiveness of the STVM intervention for improving PA behavior change. The PM+FF versus PM comparison was intended to provide insight into the potential effectiveness of the STVM intervention for improving supportive behaviors by family members and friends. The study consisted of a 7-day baseline period, followed by a 12-week intervention period. In-person assessments were conducted after the baseline period, at 6 weeks, and at 12 weeks. Participants received a US $10 gift card for each completed assessment. Family members and friends received a US $10 gift card at the end of the study.

### Setting and Recruitment of Participants

Participants were recruited from a diabetes management program (DMP) at an ambulatory care clinic of the Los Angeles County Department of Health Services, a public safety-net health care system. After routine clinic visits, DMP clinicians referred patients to a research assistant (RA) who was hired by the study and was not affiliated with the clinic. The RA explained the study to patients, screened for eligibility, and consented interested patients. Patients eligible to participate were of age 18 years or older, had a diagnosis of T2D, did not have a medical condition restricting participation in a walking program (judged by DMP clinicians before referral), preferred to speak English or Spanish, self-identified as Latino, had the ability to walk without the use of assistive devices, were available to attend 3 interviews at the clinic, did not have plans to move away from the region or be out of the country during the subsequent 3 months, and possessed a phone that could receive regular STVMs for 3 months.

Eligible participants received an OMRON HJ-321 Tri-Axis Pedometer (OMRON HEALTHCARE Co., Ltd., Japan) and were instructed to identify a family member or friend willing to participate in the study if need be. After the 7-day baseline period, participants were dismissed from the study if there were fewer than 3 consecutive days of data stored in the pedometer with at least 10 hours of self-reported pedometer use per day [30], the average daily steps exceeded 8800 (indicating sufficient PA) [31], or if they were unable to identify a family member or friend willing to participate in the study. Only participants who were continuing the study after the baseline period were assigned to a study arm.

### Intervention Procedures

Findings from our study of patient preferences were used to inform intervention components [27], which are summarized in Table 1. Participants in all study arms were encouraged to use the pedometers and walking logs to self-monitor daily steps. The walking logs contained instructions to set goals for gradually increasing daily steps during the course of 12 weeks until reaching 10,000 steps per day.

Participants in the PM and PM+FF arms received support via STVMs (at least 4 per week). On Sundays, participants received an STVM reminding them to review daily step goals and self-monitor using pedometers and walking logs. On Tuesdays and Thursdays, participants received a unique STVM with educational content largely adapted from public material available on the websites of the American Diabetes Association, National Institute of Diabetes and Digestive and Kidney Diseases, and Healthy People 2020. On Saturdays, participants received an STVM asking them to report on their perceived PA performance. If participants replied, they received another STVM providing tailored feedback. Participants in the PM+FF arm had a family member or friend receiving 2 unique STVMs per week (on Tuesdays and Thursdays) that suggested things they could do or say to support the participant’s PA behavior change efforts [32]. Unless there was a scheduling conflict, all STVMs were delivered at 9:00 AM on the aforementioned days of the week. Table 2 contains an example of each type of STVM delivered to participants and family members and friends.

**Table 1:** Intervention components for each study arm.

Intervention components		Control	PM^a^	PM+FF^b^
Self-monitoring (pedometers and walking logs)		✓	✓	✓
Goal setting (recommended 10,000 steps/day)		✓	✓	✓
**Support via STVM^c^**			✓	✓
	Reminder to review goals and self-monitor (1 STVM/week)			
	PA^d^ behavior change education (2 STVMs/week)			
	Reporting on PA performance (1 STVM/week)			
	Feedback on PA performance (1 STVM/week)^e^			
Support from a family member or friend (2 STVMs/week)				✓

^a^PM: phone messaging.

^b^PM+FF: phone messaging plus social support from family members and friends.

^c^STVM: short text or voice message.

^d^PA: physical activity.

^e^Delivered only if participant reported on PA performance.

**Table 2:** Examples of short text or voice messages.

Recipient	STVM^a^ type	Example
Participants in PM^b^ and PM+FF^c^ arms	Reminder to review goals and self-monitor PA^d^	Remember to review your daily step goals, wear your pedometer, and fill out your walking log.
	PA behavior change education	Brisk walking can lower your blood sugar and improve your A1C. Your doctor may instruct you to take fewer diabetes pills or less insulin. Brisk walking will leave you feeling better so you can do activities you enjoy, such as spending quality time with family and friends. Walk first thing in the morning before your day gets too busy. If you don’t have 30 minutes, look for three 10-minute periods.
	Reporting on PA performance	How well did you do with your daily step goals in the past 7 days? Reply with a number from 1 (not well at all) to 5 (excellent).
	Feedback on PA performance	If response was 1, 2, or 3: Walking needs to be a regular habit to produce benefits. Make an effort to improve your walking in the next 7 days. If response was 4 or 5: Great! Keep up your hard work, and you will see that it will pay off. Increase your daily goal by 1000 steps.
Family members and friends of participants in PM+FF arm	Supportive behaviors	Brisk walking can help lower the patient’s blood sugar to keep diabetes under control. Offer your support by joining them on a brisk walk as often as you can.

^a^STVM: short text or voice message.

^b^PM: phone messaging.

^c^PM+FF: phone messaging plus social support from family members and friends.

^d^PA: physical activity.

Each person receiving STVMs indicated preference for message type (voice or text) and language (English or Spanish). All messages were written in English, translated to Spanish by a native Spanish speaker, and reviewed by a second Spanish speaker for accuracy. All voice messages were voice recorded in English and Spanish by a bilingual Spanish-English speaking woman who did not otherwise have contact with participants.

### Study Measures

This study assessed technical feasibility, perceived usefulness, and potential effectiveness of the STVM intervention. Baseline, 6-week, and 12-week assessment questions are available in Multimedia Appendix 1.

#### Technical Feasibility

Technical feasibility was assessed by examining receipt of STVMs, engagement with STVMs requiring a response, barriers to receipt of and engagement with STVMs, and pedometer usability. For receipt of STVMs, data on the receipt of voice messages was obtained from the service provider that delivered the voice messages. An individual receiving voice messages was considered to have received an STVM if there was a live answer or if the message was left in a voicemail. Data on the receipt of text messages were obtained from participants’ self-report during the 6- and 12-week assessments. For engagement with STVMs, participants were considered engaged if they replied to STVMs requiring a response. For barriers to receipt of and engagement with STVMs and perceived pedometer usability, participants were asked during the 6- and 12-week assessments to explain any problems they had with receiving STVMs, replying to STVMs, or using pedometers.

#### Perceived Usefulness

Perceived usefulness was assessed by asking participants during the 6- and 12-week assessments to what extent they perceived the program to enhance their ability to make PA behavior changes. More specifically, the assessment questions inquired about participants’ perceptions of setting PA goals, self-monitoring and reporting on PA performance, receiving educational and feedback STVMs, and the idea of using STVMs to communicate with patients about PA behavior change. For participants in the PM+FF arm, the questions also inquired about supportive behaviors exhibited by family members and friends since the start of the program as well as participants’ perceptions of the idea of using STVMs to communicate with family members and friends about their PA behavior change efforts.

#### Potential Effectiveness

Potential effectiveness was assessed by measuring PA and perceived social support from family members and friends at baseline, 6 weeks, and 12 weeks. PA was used to assess the potential effectiveness of the STVM intervention for improving PA behavior change. To measure PA, average steps per day in the past 7 days were obtained from the pedometer 7-day data storage. Steps per day were considered an appropriate measure of PA in this study because the intervention specifically promoted walking. Perceived social support from family members and friends was used to assess the potential effectiveness of the STVM intervention for improving supportive behaviors by these individuals. To measure perceived social support from family members and friends, a modified version of the Social Support and Exercise Survey [32] was used. Participants were asked to evaluate how often they perceived supportive behaviors from either family members or friends. Participants responded on a Likert scale from 1 (none) to 5 (very often), with higher numbers indicating higher perceived support.

### Sample Size

The sample size was 42 total participants, 14 per study arm. It was based on a rule of thumb, which is an accepted method for setting sample sizes in pilot studies [33]. We used the rule of thumb of 12 participants per group put forth by Julious et al [34], but we increased the sample size to 14 per group to anticipate having about 12 participants per group complete the study. Our experience in conducting studies at the DMP clinic led us to expect about a 16% dropout rate.

### Randomization and Blinding

Participants were randomized with equal probability into one of the 3 study groups. A Web-based statistical computing program was used to generate a simple randomization schedule. The RA assigned participants to a study arm via opaque sealed envelopes marked according to the randomization schedule. Participants, the RA who administered all assessments, and the data analysts were not blinded.

### Analysis

To analyze participants’ qualitative responses to technical feasibility questions, the authors used the following a priori codes that directly reflected the technical feasibility measures: barriers to receipt of STVMs, barriers to engagement with STVMs, and pedometer usability. Excerpts in each code were then sorted to identify themes. The most salient themes are presented in the findings. To analyze participants’ qualitative responses to perceived usefulness questions, the authors used the following a priori codes that were derived largely from self-regulation techniques [35]: ongoing behavior change support, self-monitoring, goal setting, self-instruction, reporting on PA performance, receiving feedback, and social support. Social support was further divided into subcodes representing broad types of supportive behaviors: instrumental, emotional, and informational. Excerpts in each code were then sorted to identify themes. The most salient themes are presented in the findings. The first author coded the data independently and then discussed the coding with the second author to reach consensus for final coding. Dedoose version 7.1.3 was used to manage and code the qualitative data. Descriptive statistics are provided for receipt of and engagement with STVMs requiring a response.

To analyze potential effectiveness, participants’ average steps per day were computed for consecutive days (at least 3) wherein participants wore the pedometer for at least 10 hours per day; otherwise, their daily step counts were not included in the analysis. Participants’ perceived social support from family members and friends was computed by averaging the Likert-scale points across items in the scale. Means and standard deviations for the 3 study arms were calculated for each outcome measure. Analysis of variance was used to assess differences in means among study arms. Paired *t* tests were used to assess differences in means within study arms. *T* tests were used to assess differences in the difference in means between study arms. All analyses were conducted at the .05 significance level using STATA version 14.2.

## Results

**Figure 1:**
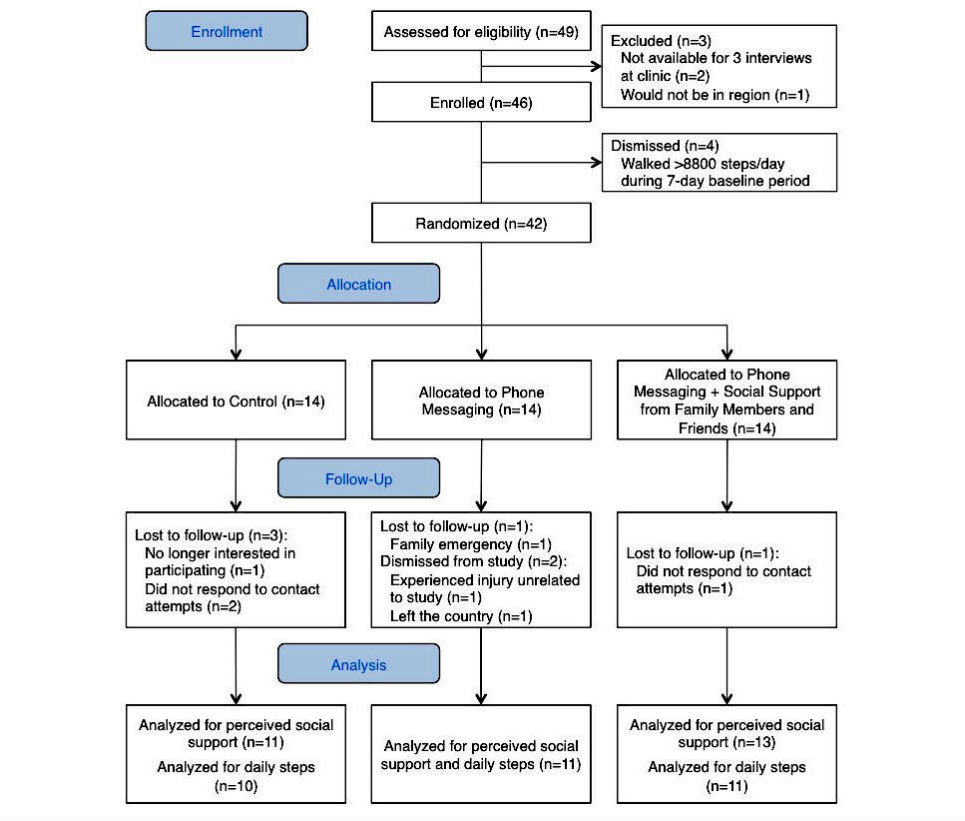
Participant flow diagram.

Participants were recruited from April to August 2015, and follow-up assessments were conducted until November 2015. Figure 1 displays the participant flow diagram. Table 3 presents participant characteristics; there were no statistically significant differences in participant characteristics among the three study arms.

**Table 3:** Participant characteristics. There were no statistically significant differences among study arms at the .05 level of significance.

Characteristics		Total (N=42)	Control (N=14)	PM^a^ (N=14)	PM+FF^b^ (N=14)
Gender (female), n (%)		28 (67)	9 (64)	7 (50)	12 (86)
Age in years, mean (SD)^c^		52 (9)	53 (8)	53 (9)	50 (9)
Spanish as preferred language, n (%)		32 (76)	10 (71)	12 (86)	10 (71)
**Educational attainment, n (%)**					
	Less than high school	22 (52)	8 (57)	5 (36)	9 (64)
	High school graduate	4 (10)	1 (7)	2 (14)	1 (7)
	More than high school	16 (38)	5 (36)	7 (50)	4 (29)
Years since T2D^d^ diagnosis, mean (SD)		12 (9)	12 (7)	11 (11)	12 (8)
Phone with text-messaging capability, n (%)		36 (86)	12 (86)	11 (79)	13 (93)
Prefer voice instead of text messages, n (%)		11 (39)		6 (43)	5 (36)

^a^PM: phone messaging.

^b^PM+FF: phone messaging plus social support from family members and friends.

^c^SD: standard deviation.

^d^T2D: type 2 diabetes.

### Technical Feasibility

In terms of receipt of STVMs and engagement with STVMs requiring a response, all participants reported receiving STVMs throughout the 12-week study. Participants receiving text messages reported receiving an average of 4 or 5 messages per week and responded to 62.7% (128/204) of messages requiring a response. Participants receiving voice messages were reached on 91.6% (308/336) of calls and provided a response during 30% (12/40) of calls that were answered and required a response.

In terms of barriers to receipt of and engagement with STVMs, 18% (3/17) of participants receiving text messages reported that they did not respond to messages asking about their PA performance because they either did not understand the instructions or because they did not know how to send text messages. Similarly, since they did not understand the instructions, 29% (2/7) of participants receiving voice messages stated that they did not respond. Participants receiving voice messages also said that they were too busy to respond (2/7, 29%), were not given enough time to respond before the call ended (1/7, 14%), or were confused after hearing an English voice on the call (default, non-customizable message giving instructions for how to opt out of calls; 3/7, 43%).

In terms of pedometer usability, 13% (3/24) of participants stated that they did not wear the pedometers consistently because the pedometers would fall off the clips; 17% (4/24) reported having difficulty reading the text on the pedometer screen and navigating the various screen display options; 13% (3/24) admitted at the end of the study that although they wore the pedometer each day, they never learned to read their steps; and 25% (6/24) stated that they were afraid of accidentally pushing the “wrong” button on the pedometer.

### Perceived Usefulness

Participants described how the program enhanced their ability to make PA behavior changes, which we categorized as providing convenient and ongoing behavior change support; prompting self-monitoring, goal setting, and self-instruction; reporting on PA performance and receiving feedback; and instrumental, emotional, and informational support. Direct quotations from participants can be found in Table 4.

#### Providing Convenient and Ongoing Behavior Change Support

Several participants in the PM and PM+FF arms explained that receiving STVMs was a better alternative than a clinic-based program because restrictive work hours and transportation issues would make it difficult for them to participate in the latter. In addition, according to participants, receiving regular STVMs was a good way of being reminded to stay active in between visits to the DMP clinic. Many participants stated that they preferred receiving text messages to talking to a real person because it was more convenient and because they felt more comfortable texting than speaking to a real person. However, an individual suggested that participants periodically receive a call from a real person.

#### Prompting Self-Monitoring

In terms of using pedometers as a self-monitoring tool, most participants in the PM and PM+FF arms viewed the devices favorably and recognized that wearing them enabled objective tracking of PA. Several participants also explained that writing their daily steps in a log allowed them to have a longer record of how much they walked because the pedometers only stored 7 days of data. According to these participants, viewing their daily steps prompted them to assess whether they were making progress toward the long-term goal of 10,000 steps per day and allowed them to discover patterns in their PA behavior.

#### Prompting Goal Setting

Most participants in the PM and PM+FF arms reported that having a daily step goal motivated them to walk more throughout the day. One participant, on the other hand, disliked goal setting because it was “traumatizing” not to meet the goal. Participants had mixed perceptions about the recommended long-term goal. Some were satisfied with the 10,000 daily step goal, others challenged themselves by setting an even greater goal, and other participants felt that the goal was too ambitious.

#### Prompting Self-Instruction

According to participants in the PM and PM+FF arms, having information about their actual PA behavior prompted them to self-instruct. After reviewing their progress, participants explained how they would reflect on how they needed to keep walking until they reached a certain step count, walk more steps the next day, or think about how walking was going to benefit their health. Participants said this type of self-instruction served as a motivation to walk more.

#### Reporting and Receiving Feedback

Participants in the PM and PM+FF arms stated that the STVMs asking them to report their PA performance prompted them to self-reflect and motivated them to improve their PA behavior in order to provide a more favorable response the next time. Participants enjoyed receiving positive feedback when they replied with a high number. Although 1 participant reported being indifferent about the feedback messages, several others reported that these messages motivated them to continue their behavior change efforts and increase their daily steps. In terms of the reporting mechanism, 1 participant liked that it was quick and easy to reply with a single number. Another participant, however, perceived this as a limitation because of the inability to explain the reason for the given response.

**Table 4:** Exemplar quotations from participants describing how they perceived the program to be useful.

Category	Exemplar
Convenient and ongoing behavior change support	“I like that I don’t have to go to the clinic to get help for physical activity because I live far.”^a^ “The doctor will tell me (during a clinic visit) to walk, but then we won’t discuss it again until the next visit. I like that the messages constantly remind me.”^a^ “If you are not home or you cannot pick up the call, the text message is saved and you can read it any time.”^a^
Self-monitoring	“Having a pedometer keeps you from lying to yourself that you did walk enough.”^a^ “I found out that on Saturdays, I walk the most—that is because I go to parties and dance a lot.”^a^ “I try to see which days I had the most steps. I want to see what kinds of things I did that day that made me get a lot of steps. I noticed that on the weekends, I don’t walk that much.”^b^
Goal setting	“(Setting a goal) is a good idea because it tells me what I need to work towards.”^b^ “I like having goals because they motivate me to walk more. Without goals, I don’t think I would walk as much as I do now.”^a^ “The 10,000 steps goal is too much. I have to go walk, then rest, then walk, then rest. My back and my legs hurt because of the arthritis.”^a^
Self-instruction	“When I don’t have enough steps, I tell myself that I need to keep walking more.”^a^ “When I don’t walk enough that day, I tell myself that I need to walk more the next day.”^a^ “I ask myself, ‘Do I want to walk more or do I want to take insulin?’”^b^
Reporting and feedback	“I like reporting because it helps me to keep track of how I am doing. Each week, I try to improve so that I will give a higher number the next time.”^a^ “It’s like someone grading you, like you did good on your test.”^b^ “I liked that it was so simple to reply, just one number. I didn’t have to type out a long response. Even if you don’t have time, you can quickly type one number.”^a^
Instrumental support	“(My husband) constantly asks me, ‘Did you walk already? If you haven’t, let’s eat dinner and then go.’ He walks with me, and I forget that I am exercising because we begin talking.”^a^ “(My husband) helps me around the house so that I have time to exercise… He tells me that he will watch our baby so that I could go walk with my sister… I feel like our relationship has improved.”^a^ “(My husband) parks his car very far so that we can walk more. He takes me to go walking because he says the text messages told him to.”^a^
Emotional support	“I like that someone is concerned and cares and takes the time to check on me. It gives me more motivation.”^b^ “I am thankful that someone was interested in my health. I have put more effort into walking more.”^a^ “The messages motivate me. I don’t have family so knowing that someone cares about me makes me feel special.”^b^
Informational support	“I didn’t exactly know why I had to exercise. I didn’t know it was beneficial for my health.”^b^ “Before (participating in this program), I didn’t know how many steps I needed to walk each day.”^b^ “My mom says, ‘You know the drill. I am going to call later to see how much you walked. Even if you don’t feel like it, just get up and go around the school a few times. Just do something, and then you feel like doing more.’”^a^

^a^Quotation from a participant in the phone messaging + social support from family members and friends (PM+FF) arm.

^b^Quotation from a participant in the phone messaging (PM) arm.

#### Instrumental Support

Most participants in the PM and PM+FF arms felt that the STVMs motivated and reminded them to be active. One person described STVMs as “an alarm to go out and walk.” The majority of PM+FF participants reported that family members and friends regularly reminded them to walk, offered encouraging words, inquired about how much they had been walking, or walked with them. Another common form of instrumental support from family members and friends was creating opportunities for participants to be more active; for example, by parking vehicles farther away from destinations or by helping with household responsibilities in order to free up time for the participant.

#### Emotional Support

Only one PM+FF participant stated that their family member or friend was a source of emotional support—that is, the family member or friend was perceived as caring about the participant’s PA behavior. On the other hand, numerous PM and PM+FF participants perceived emotional support from the receipt of STVMs from the program. They used words such as “care,” “concerned,” and “interested” to describe how the STVM messaging system “felt” about the participants’ behavior change efforts and well-being.

#### Informational Support

Several participants from both intervention arms stated that they learned from the STVMs, for the first time, about the benefits of PA for individuals with diabetes. Many participants in the PM+FF arm also reported having received this type of information from family members and friends. Additionally, these participants described how family members and friends regularly offered ideas of where and how to be active.

### Potential Effectiveness

**Figure 2:**
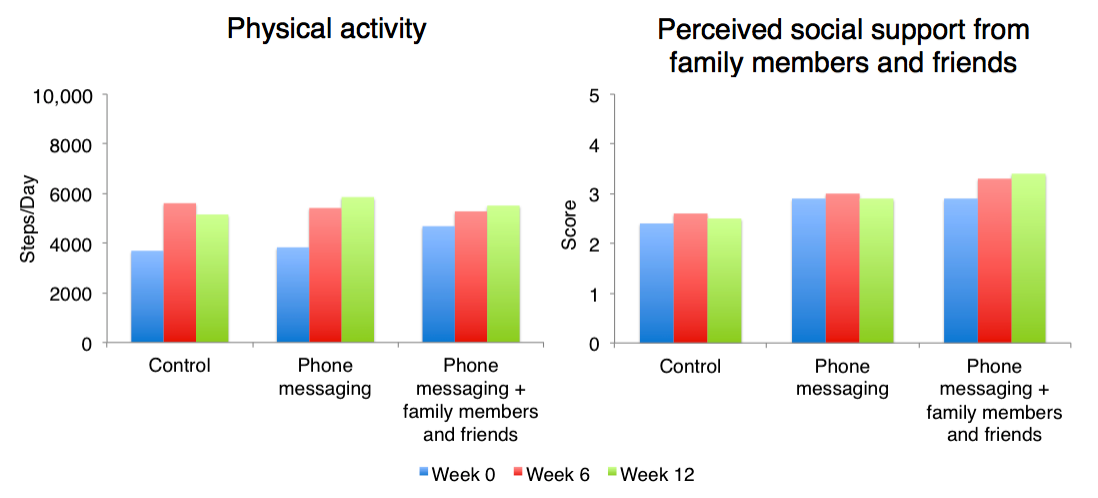
Changes over time in physical activity and perceived social support from family members and friends. Perceived social support is based on the Social Support and Exercise Survey, with higher numbers indicating higher perceived support.

PA (steps/day) and perceived social support from family members and friends for each study arm at Weeks 0, 6, and 12 are presented in Table 5 and Figure 2. There were no significant differences in outcomes among study arms at Week 0. The following sections describe differences within study arms during the 6- and 12-week follow-up assessments. Unless otherwise stated, these within study arm differences were not statistically significant. In addition, differences in differences for each outcome were not statistically significant at Week 6 or Week 12.

#### Daily Steps

Participants in all study arms increased their PA (steps/day) from Weeks 0 to 6. The increases within the control and PM arms were statistically significant. The control arm had the greatest increase, followed by the PM arm and then the PM+FF arm; however, the PM and PM+FF arms started with higher levels of PA than the control arm at baseline. Only participants in the PM and PM+FF arms continued to increase their PA from Weeks 6 to 12. The increase in PA was highest within the PM arm.

**Table 5:** Physical activity and perceived social support from family members and friends at baseline and change from the previous assessment. Values are mean (standard deviations). There were no significant differences among groups at Weeks 0, 6, and 12.

Outcome	n	Control			n	PM^a^			n	PM+FF^b^		
		Week 0	Week 6	Week 12		Week 0	Week 6	Week 12		Week 0	Week 6	Week 12
		M (SD)^c^	MΔ^d^ (SD)	MΔ (SD)		M (SD)	MΔ (SD)	MΔ (SD)		M (SD)	MΔ (SD)	MΔ (SD)
Physical activity (steps/day)	10	3691 (892)	1915 (2308) (*P*=.03)	−454 (1733)	11	3829 (1205)	1584 (1858) (*P*=.02)	439 (2069)	11	4680 (2731)	597 (2039)	233 (2538)
Perceived social support from family members and friends	11	2.4 (0.9)	0.2 (0.6)	-0.1 (0.6)	11	2.9 (0.9)	0.1 (0.8)	−0.1 (0.4)	13	2.9 (0.7)	0.4 (0.8)	0.1 (0.5)

^a^PM: phone messaging.

^b^PM+FF: phone messaging plus social support from family members and friends.

^c^SD: standard deviation.

^d^Δ: (current assessment value−previous assessment value).

#### Perceived Social Support from Family Members and Friends

Participants in all study arms had an increase in their perception of social support from family members and friends from Weeks 0 to 6. The PM+FF arm had the greatest increase, followed by the control arm and then the PM arm. Only participants in the PM+FF arm continued to have an increase in the perception of social support from family members and friends from Weeks 6 to 12.

## Discussion

### Principal Findings

This study found that it was feasible to reach and engage urban, low-income Latino adults with T2D and their family members and friends using an STVM intervention. However, receipt of and engagement with STVMs varied by mode of message delivery; participants who opted to receive text messages were reached more and were more engaged than those who opted to receive voice messages. The majority of participants complied with wearing pedometers as instructed, although several encountered barriers with wearing and using pedometers. In addition, this study found that guidance in self-regulation was a useful mechanism for supporting PA behavior change via STVMs. Specifically, participants generally perceived as useful the prompting of self-monitoring, goal setting, self-instruction, reporting and feedback, and social support. Finally, participants in all study arms improved their PA (steps/day) in the first half of the study, but only intervention participants continued to improve their PA in the second half of the study. Similarly, the perception of social support from family members and friends improved for participants in all study arms in the first half of the study. In the second half of the study, however, perceived social support improved only for participants that had a family member or friend receiving STVMs as part of the intervention.

There is evidence that Spanish-speaking adults with diabetes can be successfully engaged in a self-management intervention delivered using interactive voice response phone calls [23,24]. Participants in our study who received voice messages were willing to reply to messages requiring a response, but the platform used to deliver voice messages created barriers, which could have been prevented if the platform could be customized in terms of language and time given to respond. If using a platform with similar limitations, the results of this study suggest that text messages are superior to voice messages for ensuring patient engagement. Given that most participants reported having a phone with text-messaging capability, a larger study could restrict the mode of delivery to text messages and teach participants how to send and receive them.

Our study demonstrates that self-regulation techniques can be successfully applied in an STVM intervention to support PA behavior change and that patients perceive these techniques as useful. A recent systematic review examining the impact of information technology on behavior change for various health conditions found that less than a third of interventions delivered via phones explicitly reported using a behavior change theory as a guide for intervention design [36]. Among those that did report using behavior change theory, none used self-regulation theory even though self-regulation techniques (ie, goal setting, self-monitoring, and feedback) are associated with positive outcomes in interventions that promote PA and healthy eating [28,29]. Future studies may use our intervention components, which are aligned with self-regulation theory and patient preferences, to inform the design of phone-based interventions.

There is evidence that using a pedometer to self-monitor daily steps is associated with increases in PA [37]. Given that improvements in PA within the control arm were observed only in the first half of the study, it appears that being prompted to use a pedometer and walking log to self-monitor and being prompted to set goals was a sufficient short-term intervention. However, ongoing support via STVMs and family members and friends appeared to be a promising approach for continued improvements in PA. This finding is consistent with evidence indicating that individuals require ongoing self-management support in order to maintain initial gains achieved through intervention [5]. Furthermore, there are limited published studies examining technology-based chronic disease self-management interventions that also incorporate a component of support from family members and friends [23,24]. These studies have not examined the additional benefit, if any, on patient outcomes. Our results indicate that using STVMs to prompt supportive behaviors from family members and friends has the potential to improve perceived social support. Future research is needed to investigate how improvements in perceived social support resulting from an STVM intervention impact PA behavior.

### Limitations

The first limitation is the small sample size. The small sample size made it hard to check whether the distributional assumptions of the hypotheses tests were met. However, we re-did the analysis using nonparametric tests (not presented) and the conclusions did not change. The second limitation is that the randomization process resulted in the PM+FF arm having participants with more daily steps at baseline compared with the other arms. The results of this imbalance may explain the observation of a significant increase in daily steps in the control and PM arms, but not the PM+FF arm. Another limitation is that the majority of participants’ perceptions about the usefulness of the program were positive, which may be an indication of social desirability bias. However, only 1 participant was lost to follow-up due to not responding to our contact attempts (compared with 3 in the control arm), making us confident that our results indeed represent participants’ perceptions. A final limitation is that we measured perceived social support instead of actual social support. Participants in the PM+FF arm could have perceived higher levels of social support from family members and friends—whether or not this was actually the case—because they knew that their family members and friends were being prompted to provide support. However, in qualitative interviews, PM+FF participants provided specific examples of how the supportive behaviors of their family members and friends had changed since the start of the study, which helps to validate our quantitative results.

### Conclusions

This study demonstrated the potential of using STVMs to support PA behavior change among urban, low-income Latino adults with T2D and to prompt social support from family members and friends. Text messaging may be a better mode of message delivery than voice messaging for ensuring participant receipt of and engagement with messages. Pedometers can successfully be used by investigators for data collection purposes and by participants for self-monitoring, although adjustments to instructions are needed so that participants feel more comfortable using this tool without fear. Moreover, designing an STVM intervention based on self-regulation techniques (ie, self-monitoring, goal setting, self-instruction, feedback, and social support) is feasible and perceived as useful by participants. Finally, such an intervention may improve PA in terms of daily steps and perceived social support from family members and friends who participate in the intervention.

## Appendix

Multimedia Appendix 1 Assessment questions.

## Abbreviations

DMP: diabetes management program
PA: physical activity
PM: phone messaging
PM+FF: phone messaging plus social support from FF
RA: research assistant
SCT: Social Cognitive Theory
STVM: short text or voice message
T2D: type 2 diabetes
