# Supplementary material for: Phone Messaging to Prompt Physical Activity and Social Support Among Low-Income Latino Patients With Type 2 Diabetes: A Randomized Pilot Study
Source: JMIR Diabetes. 2017 Jun 6;2(1):e8. doi: 10.2196/diabetes.7063 (PMC6238833; doi:10.2196/diabetes.7063)
Supplement: Multimedia Appendix 1 [file diabetes_v2i1e8_app1.pdf]

Appendix: Assessments

Social Support and Exercise Survey (All participants)

I will read to you things people might do or say to someone who is trying to exercise regularly.

During the past 3 months, how often have family members (or members of my household) or friends…	Never	Rarely	A few times	Often	Very often
**1.** Exercised with you?					
**2.** Offered to exercise with you?					
**3.** Gave you helpful reminders to exercise ("Are you going to exercise tonight?")?					
**4.** Gave you encouragement to continue your efforts to exercise?					
**5.** Changed their schedule so they could exercise with you?					
**6.** Discussed exercise with you?					
**7.** Complained about the time you spent exercising?					
**8.** Criticized or made fun of you for exercising?					
**9.** Gave you rewards for exercising (bought you something or gave you something you liked)?					
**10.** Planned for exercise on recreational outings?					

During the past 3 months, how often have family members (or members of my household) or friends…	Never	Rarely	A few times	Often	Very often
**11.** Helped plan activities around your exercise?					
**12.** Asked you for ideas on how *they* can get more exercise?					
**13.** Talked about how much they liked to exercise?					

Semi-Structured Interview Questions (PM and PM+FF only)

14. Over the past 6 weeks, you should have been receiving telephone calls/text messages about walking as a form exercise. Have you been receiving these telephone calls/text messages?

☐ Yes

☐ No

15. How many times per week would you say you receive telephone calls/text messages from this study? ______ times per week

16. What are your thoughts on the number of messages you receive per week?

17. What are your thoughts on the times of the day or the days of the week you receive the telephone calls/text messages?

18. What device do you use to receive these telephone calls/text messages?

☐ Landline telephone

☐ Cell phone

☐ Other: ________________________

19. Are you having any problems receiving/hearing/reading the telephone calls/text messages? Please explain.

20. What do you do when you get a message? Do you answer the call (if voice)? Do you read the message then, or later (if text)?

21. About once per week, you should have been receiving a message asking you to rate how well you did with your walking goals in the last 7 days. Have you ever received these messages?

☐ Yes

☐ No

☐ Other: ___________________________

   a. If Yes to #60: Do you usually reply to these messages?

      ☐ Yes

      ☐ No

      ☐ Other: ________________________________

   a. If No to #60a: Why?

22. How do you feel about brisk walking as a form of exercise?

23. Do you think that participating in the study has affected the amount of walking that you do? If so, how?

24. What are your thoughts on setting goals for walking?

25. How do you feel about keeping track of your walking with the pedometer and walking log?

26. Overall, what do you think about the voice/text messages you have received?

27. What do you think about the messages you received each week after reporting how you did with your goals?

28. Overall, what do you think about using telephone calls/text messages to communicate with patients about their physical activity?

29. PM+SS patient only: I understand that your family member/friend also receives telephone calls/text messages from this study as well. Do you know if your family member/friend has indeed been receiving these?

30. PM+SS patient only: What has your family member/friend told you about the telephone calls/text messages he/she receives from this study?

31. PM+SS patient only: Since the start of this study, how has the support you feel from this person for improving your walking? What does this person do or say differently to support you?

32. PM+SS patient only: Overall, what do you think about using telephone calls/text messages to communicate with patients' family members/friends about physical activity?

33. PM+SS patient only: Is there anything else you would like to tell me about your family member/friend's involvement in the study?

34. How easy or hard was it to receive telephone calls/text messages from this study?

35. How easy or hard was it to reply to telephone calls/text messages?

36. Can you tell me about your experience with wearing a pedometer?

37. Overall, what do you like the most and least about this study? What do you like the most and least about receiving telephone calls/text messages from this study?

38. If the program being tested in this study was offered to all patients as a part of their diabetes care, would you willing to participate?

39. Would you recommend the program to other patients? Please explain.

40. If you could change one thing about the program, what would it be?

41. Is there anything else you would like to tell us about your experience in this program?
